# pMIIND-an MPI-based population density simulation framework

**DOI:** 10.1186/1471-2202-14-S1-P362

**Published:** 2013-07-08

**Authors:** Marc de Kamps, David Sichau

**Affiliations:** 1 School of Computing, University of Leeds, Leeds, WY, UK; 2 LAV, Institute of Energy Technology, ETH Zurich, 8092 Zurich, Switzerland

## 

MIIND [1] is the first publicly available implementation of population density algorithms. Like neural mass models, they model at the population level, rather than that of individual neurons, but unlike neural mass models, they consider the full neuronal state space. The central concept is a population density, a probability distribution function that represents the probability of a neuron being in a certain part of state space. Neurons will move through state space by their own intrinsic dynamics or driven by synaptic input. When individual spikes do not matter but only population averaged quantities are considered, these methods outperform direct simulations using neuron point models by a factor 10 or more, whilst (at the population level) producing identical results to simulations of spiking neurons. This is in general not true for neural mass models. Population density methods also relate closely to analytic evaluations of population dynamics.

The evolution of the density function is given by a partial differential equation (PDE). In [3] a generic method was presented for solving this equation efficiently, both for small synaptic efficacies (diffusion limit; the PDE becomes a Fokker-Planck equation) and for large ones (finite jumps). We demonstrated that for leaky-integrate-and-fire (LIF) neurons this method reproduces analytic results [1] and uses of the order of 0.2 s to model 1s simulation time of infinitely large population of spiking LIF neurons.

We now have developed this method to apply to any 1D neuron point model [3], not just LIF neurons and demonstrated the technique on quadratic-integrate-and-fire neurons. We are therefore in the position to model large heterogeneous networks of spiking neurons very efficiently. A potential bottleneck is MIIND's serial simulation loop.

We developed an MPI implementation of MIIND's central simulation loop starting from a fresh code base, and addressed serialization, which is now done at the level of individual cores. Central assumption in the set up is that firing rates are communicated, not individual spikes, so bandwidth requirements are low. Latency is potentially a problem, but with the use of latency hiding techniques good scalability for up to 64 cores has been achieved ondedicated clusters. The scalability was verified with a simple model of cortical waves in a hexagonal network of populations with balanced excitation-inhibition. pMIIND is available on Sourceforge, through its git repository: git://http://miind.sourceforge.net A CMake-based install procedure is provided. Since pMIIND is set up as a C++ framework, it is possible to define one's own algorithms and still take advantage of the MPI-based simulation loop.
